# Effects of electroconvulsive therapy on cortical thickness in depression: a systematic review

**DOI:** 10.1017/neu.2024.6

**Published:** 2024-02-12

**Authors:** Tommaso Toffanin, Giulia Cattarinussi, Niccolò Ghiotto, Marialaura Lussignoli, Chiara Pavan, Luca Pieri, Sami Schiff, Francesco Finatti, Francesca Romagnolo, Federica Folesani, Maria Giulia Nanni, Rosangela Caruso, Luigi Zerbinati, Martino Belvederi Murri, Maria Ferrara, Giorgio Pigato, Luigi Grassi, Fabio Sambataro

**Affiliations:** 1 Department of Neuroscience and Rehabilitation, Institute of Psychiatry, University of Ferrara, Ferrara, Italy; 2 Department of Neuroscience (DNS), University of Padova, Padua, Italy; 3 Padova Neuroscience Center, University of Padova, Padua, Italy; 4 Department of Psychological Medicine, Institute of Psychiatry, Psychology and Neuroscience, Kings College London, London, UK; 5 Department of Medicine, University of Padova, Padua, Italy; 6 Department of Psychiatry, Padova University Hospital, Padua, Italy

**Keywords:** ECT, cortical thickness, depression, Hamilton rating scale for depression, treatment-resistant depression

## Abstract

**Objective:**

Electroconvulsive therapy (ECT) is one of the most studied and validated available treatments for severe or treatment-resistant depression. However, little is known about the neural mechanisms underlying ECT. This systematic review aims to critically review all structural magnetic resonance imaging studies investigating longitudinal cortical thickness (CT) changes after ECT in patients with unipolar or bipolar depression.

**Methods:**

We performed a search on PubMed, Medline, and Embase to identify all available studies published before April 20, 2023. A total of 10 studies were included.

**Results:**

The investigations showed widespread increases in CT after ECT in depressed patients, involving mainly the temporal, insular, and frontal regions. In five studies, CT increases in a non-overlapping set of brain areas correlated with the clinical efficacy of ECT. The small sample size, heterogeneity in terms of populations, comorbidities, and ECT protocols, and the lack of a control group in some investigations limit the generalisability of the results.

**Conclusions:**

Our findings support the idea that ECT can increase CT in patients with unipolar and bipolar depression. It remains unclear whether these changes are related to the clinical response. Future larger studies with longer follow-up are warranted to thoroughly address the potential role of CT as a biomarker of clinical response after ECT.

## Significant outcomes

- This review summarises how ECT affects CT in patients with unipolar or bipolar depression.
- The areas that were predominantly affected by ECT were temporo-insular and frontal regions. An association between the antidepressant effect of ECT and CT changes was reported by half of the included studies.
- Identifying the possible cortical changes associated with the clinical efficacy of ECT opens new targets to ameliorate ECT protocols.

## Limitations

- The review is based on studies with small numbers of patients and considerable heterogeneity in terms of patients’ characteristics and ECT protocols, thus reducing the strength of evidence supporting a causal link between ECT and CT changes.

## Introduction

Electroconvulsive therapy (ECT) was first used in 1938 by Italian scientists Ugo Cerletti and Lucio Bini to induce an epileptic seizure to treat patients with schizophrenia (Gazdag and Ungvari, 2019). In the following decades, the introduction of general anesthesia and accurate operational protocols greatly improved ECT techniques (Kadiyala and Kadiyala, 2018; Kellner *et al*., 2020), allowing ECT to evolve to become a promising and well-tolerated treatment. Nowadays, it is considered the safest procedure performed under general anesthesia (Tørring *et al*., 2017). ECT is an important therapeutic strategy for affective disorders, including severe major depressive disorder (MDD), particularly with psychotic symptoms and a high risk of suicide, treatment-resistant depression (TRD), and depression in bipolar disorder (BD) (Kho *et al*., 2003; Fornaro *et al*., 2020). Despite the major advances in psychopharmacology and brain stimulation techniques, ECT remains one of the most effective treatments in psychiatry, with response rates in the treatment of unipolar and bipolar depression ranging from 74 to 77 % (Bahji *et al*., 2019).

The exact pathophysiological mechanisms underlying depression have not yet been clarified. Among the putative mechanisms, the “neurotrophin hypothesis of depression” suggests that a stress-induced decrease in the expression of brain-derived neurotrophic factor (BDNF) leads to atrophy of stress-vulnerable neurons in the hippocampus (Duman *et al*., 1997). Later studies expanded this theory, indicating that changes in BDNF expression and function result in structural brain alterations not only in the hippocampus, but also in the prefrontal cortex (PFC) (Duman and Li, 2012). Crucially, magnetic resonance imaging (MRI) investigations have been supporting this theory, demonstrating reductions in grey matter (GM) volumes in MDD in the orbitofrontal cortex (OFC), ventromedial PFC, inferior parietal gyrus, as well as in the hippocampus, parahippocampal gyrus, insula, thalamus, basal ganglia, anterior cingulate cortex (ACC), and posterior cingulate cortex (PCC) (Koolschijn *et al*., 2009; Kempton *et al*., 2011; Wise *et al*., 2017). Similarly, a recent meta-analysis of volumetric studies in MDD identified decreased GM volume in frontal, temporal, and limbic regions. Interestingly, the authors also found an association between drug treatment and larger GM volume in the right striatum and smaller GM volume in the right precuneus (Jiang *et al*., 2023).

In bipolar depression, similar volumetric alterations have been reported, particularly in the frontal and limbic areas. In addition to volumetric changes, alterations in surface measurements have also been reported in depression, such as cortical thickness (CT), which represents the depth of GM as the covering neural sheet of brain foldings and is correlated with the size of neurons, neuroglia, and nerve fibres (Narr *et al*., 2005). Importantly, a reduction in CT could indicate a reduction in dendritic arborisation or changes in myelination at the grey/white matter interface (Sowell *et al*., 2004) The Enhancing Neuroimaging Genetics through Meta-Analysis (ENIGMA) Consortium has demonstrated that lower CT in temporal regions is a common feature in various neuropsychiatric disorders (Schmaal *et al*., 2017; Boedhoe *et al*., 2018; Van Rooij *et al*., 2018; Hoogman *et al*., 2019). With regards to MDD, the ENIGMA Major Depressive Disorder Working Group reported thinner cortical GM in MDD compared to controls in the OFC, ACC and PCC, insula, and temporal lobes (Schmaal *et al*., 2017). Furthermore, a decrease in CT was also found in elderly patients with MDD (Lim *et al*., 2012) and in those with a high risk for suicide (Wagner *et al*., 2012) Additionally, a thinner CT in the PCC could indicate a higher risk of non-remission in MDD patients (Järnum *et al*., 2011). In BD, a recent ENIGMA study highlighted that individuals with BD showed a widespread thinner cortex relative to HC, with the largest effect size in the left fusiform gyrus (Matsumoto *et al*., 2023).

Although the association between surface measures and the severity of depressive symptoms is unclear, changes in CT after response and remission have been reported after antidepressant treatment. In particular, in a 12-week trial using sertraline in patients with late-life depression, nonremitters presented thinner frontal poles at baseline compared to remitters (Sheline *et al*., 2012). Another trial showed that sertraline increased CT in the left medial PFC, right medial and lateral PFC, and within the right parietal-temporal lobes in patients with MDD compared to placebo (Nemati and Abdallah, 2020). In addition, CT also predicted the effectiveness of psychotherapy for late-life depression (Mackin *et al*., 2013). Therefore, reduced CT appears to be related to the reduced efficacy of pharmacological interventions in MDD, and successful treatment appears to reverse atrophy in fronto-limbic areas (Motter *et al*., 2021). Crucially, reduced CT appears to be associated also with early-life traumas and with other affective disorders, including BD and anxiety (Zhu *et al*., 2022; Yang *et al*., 2023).

The neural mechanisms underlying the effects of ECT in the treatment of depression are still largely unknown, and this further contributes to the scepticism about its use (Bolwig, 2011). Importantly, recent meta-analytic evidence suggested that ECT is associated with volume increases in the hippocampus and increased integrity of white matter pathways in the frontal and temporal lobes (Gbyl and Videbech, 2018). These findings have also been confirmed by a mega-analysis conducted by the Global ECT-MRI Research Collaboration (GEMRIC), which showed that ECT induced broadly distributed GM volume increases (Ousdal *et al*., 2020). GM volume is determined by CT and surface area; nevertheless, the relationship between GM volume and CT is modest, both globally and locally (Winkler *et al*., 2010). Conversely, GM volume is highly correlated with surface area. Their limited relationship most probably depends on unrelated genetic factors that are likely tied to different neurobiological processes (Winkler *et al*., 2010), and is further biased by grey/white matter intensity contrast and curvature differences (Kong *et al*., 2015). For this reason, we wanted to study changes in CT after ECT treatment with the expectation that these results would be complementary to those reported in cortical GM volumetric studies. Given these premises, we aimed to review the available evidence on CT alterations associated with ECT treatment in patients with depression to explore the association between changes in CT and the antidepressant effect of ECT.

## Materials and methods

### Literature search and screening

The study inclusion criteria were the following: a) participants with a diagnosis of MDD or BD according to the Diagnostic and Statistical Manual of Mental Disorders (DSM) or International Classification of Disease (ICD) criteria or TRD (any definition); b) participants underwent MRI pre- and post-ECT; c) changes of CT were assessed longitudinally, that is, before and after the ECT treatments; c) CT changes were longitudinally evaluated, that is, before and after the ECT sessions (no restrictions in terms of the number of sessions or temporal distance between ECT and MRI).

A comprehensive search of the literature was performed on PubMed, Medline, and Embase using the following keywords: (ECT [Title/Abstract] OR Electroconvulsive Therapy [Title/Abstract] OR Electroconvulsive Treatment [Title/Abstract]) AND ((Major Depressive Disorder [Title/Abstract] OR Depression [Title/Abstract] OR Bipolar Disorder [Title/Abstract**]**) OR Bipolar Depression [Title/Abstract]) AND Cortical thickness [Title/Abstract]). No language restrictions were applied to identify peer-reviewed articles published up to April 2023.

**Figure 1.**
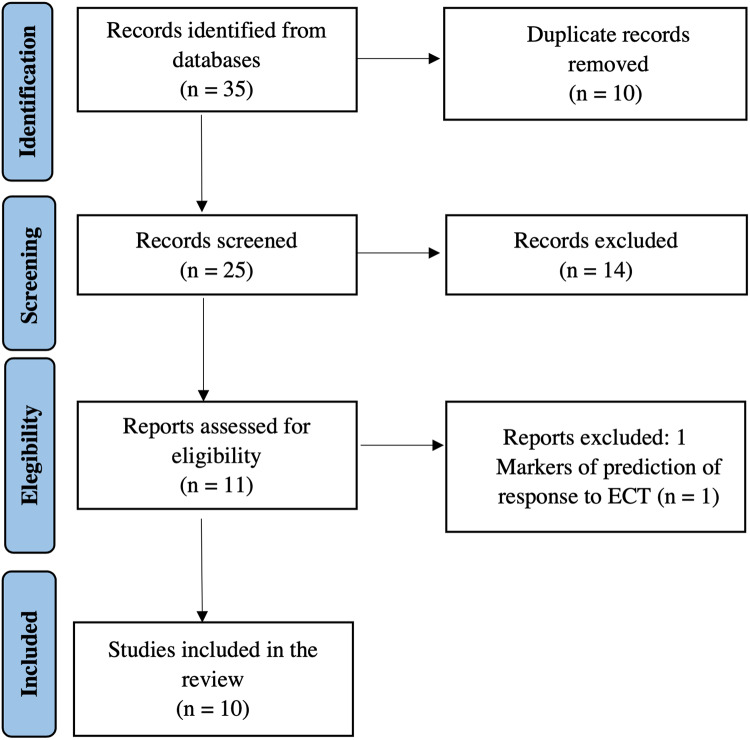
PRISMA flow chart of selection of publications for inclusion in the review.

We screened titles and abstracts of all potentially eligible studies, retrieving the full-text articles. References cited by each study were also manually screened to ensure that no relevant studies were left out. This procedure was carried out according to the Preferred Reporting Items for Systematic Reviews and Meta-Analysis (PRISMA) guidelines (Liberati *et al*., 2009). Of the 35 initial studies, 25 remained after removing duplicates, and 11 citations met the title/abstract screening eligibility criteria and were included. After full-text screening, 1 article was excluded because it did not report longitudinal changes after ECT (Wade *et al*., 2017), resulting in 10 eligible studies (Fig. 1). Since only two studies reported quantitative measures of the effects of ECT on CT, we could not perform a meta-analysis.

### Assessment of methodological quality

The methodological quality of the included studies was assessed using a point system derived from Gbly and Videbech (Gbyl and Videbech, 2018). Briefly, the quality score consisted of the following items: a) number of subjects; b) presence of a control group; c) number of MRI scans the control group underwent; d) MRI scanner field strength; e) voxel size of the MRI scan; f) medication status; g) consecutively collected sample; h) duration of follow-up. A higher score indicated better methodological quality (see Supplementary Material).

## Results

### Demographic, clinical, and ECT characteristics of included studies

The characteristics of the included studies are described in Table 1. A total of 253 patients (244 with MDD or TRD, 9 with bipolar depression, mean age 46.3 ± 10.6 years; 67.9 % female) and 143 healthy controls (mean age 48.9 ± 11.2 years; 51.7 % female) were included. The diagnosis of MDD was made according to the DSM-IV, DSM-IV-TR, and DSM-5. Depressive symptoms were assessed with the Hamilton depression rating scales (HAM-D) (Hamilton, 1960), the Montgomery–Åsberg depression rating scales (MADRS) (Montgomery and Asberg, 1979), and the Beck Depression Inventory (BDI-II) (Beck *et al*., 1996 (see reference list)). Baseline HAM-D scores ranged from 21.4 ± 5.3 to 31.8 ± 8.2, indicating moderate to severe depressive symptoms (Zimmerman *et al*., 2013) (see Supplementary Material). Three studies (Pirnia *et al*., 2016; Gbyl *et al*., 2019; Xu *et al*., 2019) reported the mean illness duration, which ranged from 70.35 ± 83.27 months to 17.7 ± 11.93 years. In three investigations, some of the included patients also presented psychotic symptoms (van Eijndhoven *et al*., 2016; Gryglewski *et al*., 2019; Schmitgen *et al*., 2020). Patients were excluded if they presented neurological disorders, substance use disorders, schizophrenia spectrum disorders and personality disorders, a history of head injury resulting in unconsciousness for more than 5 minutes, MRI contraindications, and previous ECT sessions received in the last six months/year. Depressed participants received medications at study entrance in seven studies (Sartorius *et al*., 2016; van Eijndhoven *et al*., 2016; Gbyl *et al*., 2019; Gryglewski *et al*., 2019; Xu *et al*., 2019; Schmitgen *et al*., 2020; Bracht *et al*., 2023); in most studies, the medications were kept unchanged before ECT (Gbyl *et al*., 2019; Gryglewski *et al*., 2019; Xu *et al*., 2019; Schmitgen *et al*., 2020; Bracht *et al*., 2023), while in two studies the medications were tapered (Sartorius *et al*., 2016; van Eijndhoven *et al*., 2016).

**Table 1.** Studies investigating the effect of ECT on CT in depressed patients

Author, year	Subjects (M/F)	Diagnosis	Mean age mean (SD)	Clinical scales	Age of onset/Illness duration mean (SD)	Electrode placement	N° of sessions	Field strength	Timing of MRI scans	Timing of collection of clinical scales	ECT effect on CT	Correlations between CT changes and clinical variables	Total quality score
Pirnia *et al*., 2016	Patients: 29 (11/18)	MDD-TRD (24), BD (5)	41.0 (13.0)	HAM-D, MADRS	Age of onset: 23.89 (13.57) years Illness duration: 17.15 (11.93) years	Right unilateral	11.2	1.5 × ST	- 24 hours before the first ECT - within 48 hours after the second ECT - within 1 week of the last ECT	- 24 hours before the first ECT - within 48 hours after the second ECT - within 1 week of the last ECT	Patients vs. HC (baseline): ↓ CT in the fusiform gyrus and superior temporal gyrus. Patients: ↑ CT in bilateral anterior cingulate cortex, right parahippocampal gyrus, superior temporal gyrus and temporal pole between baseline and the last scan. HC: no CT changes across time.	Negative correlation between changes in CT in the fusiform, superior and inferior temporal gyrus and HAM-D scores. In the ACC, only responders showed a correlation between CT change and symptoms improvement.	6.9
	HC: 29 (13/16)		40.1 (12.4)		NA				- two time points with intervals equivalent to patients’ first and third assessments	NA			
Sartorius *et al*., 2016	Patients: 18 (9/9)	MDD-TRD	51.7 (13.9)	HAM-D	NR	Right unilateral	11.3 ± 4.8	>2.5 ST	- 1 or 2 days before the first ECT - at least 2 days and within 2 weeks after the last ECT session	- 1-2 days before the first ECT - at least 2 days after ECT - within 2 weeks after the last ECT	Patients vs. HC: ↑ CT in right temporal pole and bilateral insula.	NR	3.8
	HC:36 (18/18)		51.6 (13.4)		NA				NR	NA			
Van Eijndhoven *et al*., 2016	Patients: 23 (8/15) [Psychotic symptoms (6)]	MDD-TRD	50.7 (8.5)	HAM-D	Age of onset: 40.8 (10.9) years	Bilateral at the temporal window	At least 10	1.5 × ST	- 1 week before the first ECT - within 1 week after the last ECT	- 1 week before the first ECT - within 1 week after the last ECT	Patients vs. HC (baseline): no differences in CT. Patients: ↑ bilateral CT in temporal lobe, inferior and middle cortex and insula.	Negative correlation between change in mean CT of the right insula and HAM-D scores. Positive correlation between change in mean CT of the left temporal pole, the left middle temporal cortex and the left inferior temporal cortex and the number of ECT sessions.	5.3
	HC: 22 (8/14)		50.8 (8.8)		NA				- two time points with intervals equivalent to patients’ first and third assessments	NA			
Gbyl *et al*., 2019	Patients: 18 (8/10)	MDD-TRD	46.8 (14.6)	HAM-D	Age of onset: 37.1 (11.6) years Illness duration: 10.1 (11.1) years	Bitemporal	Variable	>1 ST	- before the first ECT - right after the first ECT - 6 months the last ECT	- before the first ECT - right after the first ECT - 6 months after the last ECT	↑ CT in frontal, temporal and insular cortex regions immediately after the first series of ECT, which returned to baseline 6 months after the last ECT.	Negative correlation between increase in CT of the right lateral orbitofrontal cortex and HAM-D scores. No correlations between change in CT and cognitive functioning in any of the 26 tested regions.	4.8
Gryglewski *et al*., 2019	Patients: 14 (3/11) [Psychotic symptoms (3)]	MDD-TRD	46.9 (8.1)	HAM-D	NR	Right unilateral	A minimum of 8	3 ST	- twice before the first ECT - at least 24 hours after the last ECT	- twice before a series of ECT - once after a series of ECT	↑ CT after ECT in the right insula, supramarginal gyrus, inferior parietal cortex, superior temporal gyrus, inferior temporal gyrus, superior temporal sulcus, temporal pole, postcentral gyrus and fusiform gyrus.	No correlations between CT and HAM-D score.	2.4
Schmitgen *et al*., 2020	Patients: 12 (4/8) [Psychotic symptoms (5)]	MDD-TRD	46.3 (11.3)	HAM-D	NR	Right unilateral	Variable	2.5 ST	- within 5 days before the first ECT - 6-8 days after the last ECT	- within 5 days before the first ECT - 6-8 days after the last ECT	Patients vs. HC: no significant differences in CT.	Pre-ECT: correlation between changes in HAM-D scores and the CT of the left rostral anterior cingulate gyrus. Post-ECT: correlation between changes in HAM-D scores and the CT of the left rostral anterior cingulate gyrus (strength pre- to post-ECT ↓), left medial orbitofrontal gyrus (strength pre- to post-ECT ↑).	3.2
	HC: 12 (4/8)		43.5 (11.5)		NA				- one time point	NA			
Xu *et al*., 2019	Patients: 23 (11/12)	MDD-TRD	38.7 (11.0)	HAM-D	Illness duration: 70.35 (83.27) months	Bifrontal	7.3 ± 2	NR	- 12/24 hours before the first ECT; - 24/72 hours after the last ECT	- 12-24 hours before the first ECT; - 24 -72 hours after the last ECT	↑ vertex-wise CT in left temporal pole, left fusiform gyrus, left inferior parietal gyrus, left insula, right lateral occipital gyrus, right supramarginal gyrus, right superior temporal gyrus, right insula and right precentral. ↑ regional CT in bilateral fusiform gyrus, bilateral parahippocampus and inferior temporal gyrus, left superior temporal gyrus, left inferior parietal gyrus, left insula, left superior temporal sulcus, right superior frontal gyrus and right rostral anterior cingulate. ↑ vertex-wise and regional CT in left temporal pole, fusiform gyrus and bilateral insula.	Positive correlation between delayed recall of ALVT after ECT and changes in CT in the left inferior parietal gyrus. Positive correlation between the changes in vertex-wise CT in the right lateral occipital gyrus and the changes in the HAM-D scores. Negative correlation between the changes in vertex-wise CT in the right precentral gyrus and the changes in the ALVT delayed recall scores.	3.3
Yrondi *et al*., 2019	Patients: 17 (NR)	MDD-TRD	59.2 (7.1)	HAM-D	NR	Bilateral	12.1	1.5 to 2 × ST	- within 48 hours before the first ECT - after the first ECT considered effective - within 1 week of the last ECT	- within 48 h before the first ECT - after the first ECT considered effective - within 1 week of the last ECT	Patients vs. HC (baseline): ↓ CT in frontal lobe, fusiform gyrus, inferior, middle and superior temporal gyrus, parahippocampal gyrus and transverse temporal gyrus. Patients vs. HC (longitudinal): ↑ CT in superior temporal gyrus from the first and the second scan to the third scan.	No correlations between structural changes and clinical variables.	4.7
	HC: 24 (NR)		61.9 (6.9)		NA								
Ji *et al*., 2022	Patients: 56 (12/44)	MDD-TRD	40.8 (12.1)	HAM-D	Age of onset: 35.7 (11.3) years	Bifrontal	8.4 ± 1.4	>1 ST Costant current 0.9 A	- before ECT-index series - after ECT-index series	-baseline -after ECT	↑ CT in bilateral medial areas of the superior temporal gyrus and the bilateral insula.	No correlations between CT changes and HAM-D scores.	8.6
	Patients: 40 (13/27)		39.3 (12.1)		Age of onset: 33.7 (12.7) years		7.8 ± 1.4						
Bracht *et al*., 2023	Patients ECT: 20 (12/8)	MDD-TRD (16), BD (4)	44.9 (12.0)	HAM-D, BDI-II	NR	Right unilateral (*n* = 17), bitemporal (*n* = 2), bifrontal (*n* = 1)	12.7 ± 4	5 ST for right unilateral 2 ST for bitemporal or bifrontal	- before ECT-index series - after ECT-index series	- before ECT-index series - after ECT-index series	Group × time interaction patients ECT vs. patients TAU vs. HC: ↑ global mean CT in the ECT-group and in TAU-responders but not in HC.	No correlations between CT changes and clinical variables.	6
	Patients TAU: 20 (6/14)	MDD-TRD	44.2 (15)		NR				-baseline - once remission was achieved or within 12 weeks after the first MRI in case of non-remission	- every 4 weeks			
	HC: 20 (12/8)	NA	43.6 (14)		NA				-baseline - 61.2 ± 17 days after the first scan	NA			

AVLT: Auditory Verbal Learning Test; BD: bipolar disorder; BDI-II: Beck Depression Inventory-II; CT: cortical thickness; ECT: electroconvulsive therapy; HAM-D: Hamilton Rating Scale for Depression; HC: healthy controls; MADRAS: Montgomery-Åsberg Depression Rating Scales; MDD: major depressive disorder; NA: not applicable; NR: not reported; ST: seizure threshold TAU: treatment as usual; TRD: treatment-resistant depression.

Total quality score was calculated using a point system derived from Gbly and Videbech (2018) (see Supplementary Material).

**Figure 2.**
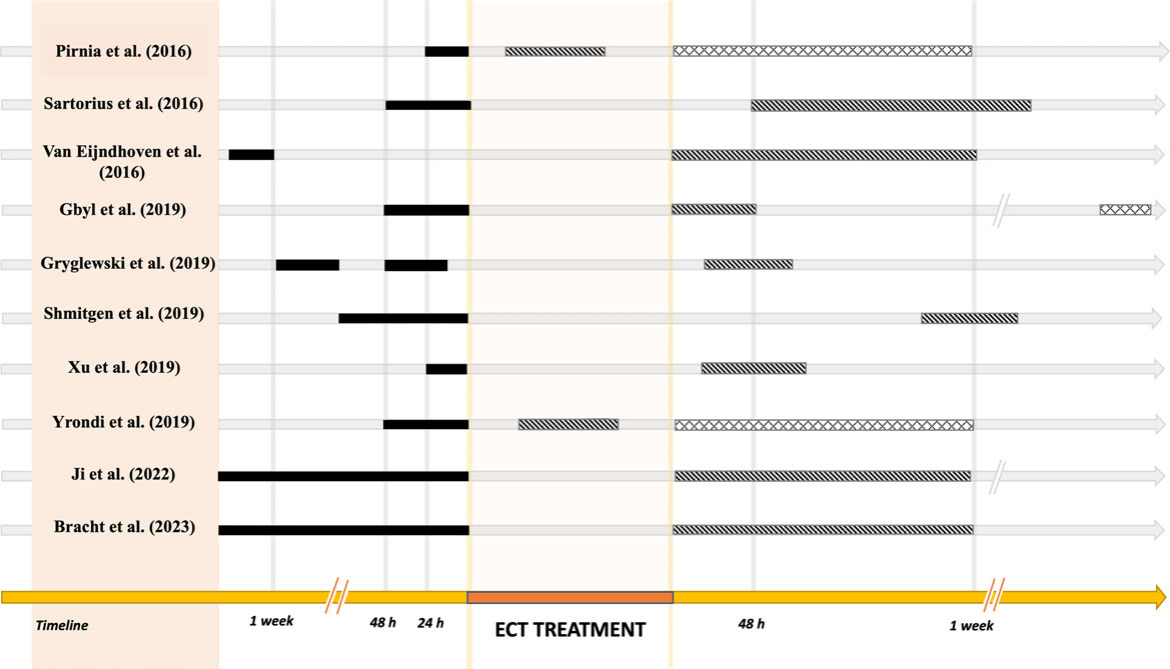
Magnetic resonance imaging (MRI) scan timeline of the studies of the effect of ECT on cortical thickness in patients with depression. For each study, we have reported the timing of the structural magnetic resonance images relative to the entire ECT treatment (in orange), including the baseline scan (solid black line, T1), the second scan (hatched line, T2), and the third scan (cross-hatched line, T3). The time is measured relative to the beginning (T1), during (T2) and after (T2, T3) the ECT treatment.

Six studies included a comparison with a group of healthy participants who did not undergo ECT treatment, while Bracht *et al*. (2023) also included an MDD group who received Treatment As Usual (TAU) (Bracht *et al*., 2023). Six studies repeated structural analysis twice (Sartorius *et al*., 2016; van Eijndhoven *et al*., 2016; Xu *et al*., 2019; Schmitgen *et al*., 2020; Ji *et al*., 2022; Bracht *et al*., 2023) and four studies performed three MRI scans (Pirnia *et al*., 2016; Gbyl *et al*., 2019; Gryglewski *et al*., 2019; Yrondi *et al*., 2019). In all studies, the first scan was acquired within a week before the ECT session and the second within a week after treatment. Two studies acquired an intermediate scan between the first and last ECT sessions to explore acute changes in CT (Pirnia *et al*., 2016; Yrondi *et al*., 2019), while one study acquired a scan before the first ECT session, a scan after the last ECT treatment, and a scan within six months after ECT treatment to examine the long-lasting effects of ECT on CT (Gbyl *et al*., 2019) (Fig. 2). Regarding the ECT protocols, five studies used bilateral electrode placement (van Eijndhoven *et al*., 2016; Gbyl *et al*., 2019; Xu *et al*., 2019; Yrondi *et al*., 2019; Ji *et al*., 2022), four studies used unilateral electrode placement (Pirnia *et al*., 2016; Sartorius *et al*., 2016; Gryglewski *et al*., 2019; Schmitgen *et al*., 2020). The study by Bracht *et al*. (2023) used mixed protocols: 17 patients started with right unilateral ECT, two patients with bitemporal ECT, and one patient with bifrontal stimulation (Bracht *et al*., 2023).

### CT changes after ECT

**Figure 3.**
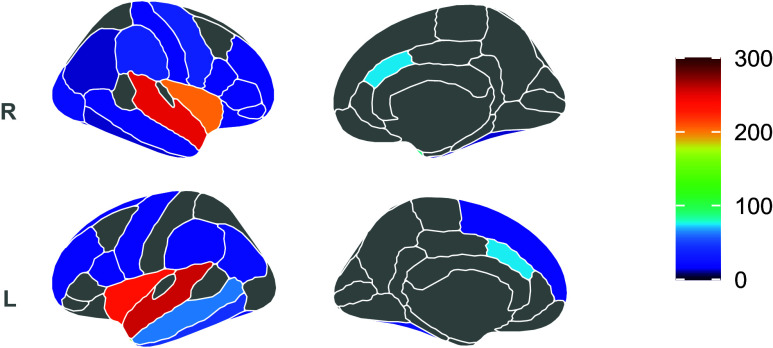
Changes in cortical thickness in patients with depression after ECT. Results from morphometric studies are displayed on the Desikan-Killiany atlas. The medial and lateral cortical surfaces are displayed for each hemisphere on the leftmost part, the subcortical regions in the center, and the colorbar on the rightmost part of each panel, respectively. The color bar code indicates the total number of subjects who showed an increase in cortical thickness. L, left hemisphere; R, right hemisphere. The renderings were created using the R-package *ggseg*.

Most studies identified CT alterations in the temporal, insular, and frontal areas, mainly proximally to the electrode (Fig. 3). Gryglewski *et al*. (2019) and Sartorius *et al*. (2016) combined voxel-based morphometry and CT analysis (Sartorius *et al*., 2016; Gryglewski *et al*., 2019). The first investigation reported a widespread increase in CT of the right hemisphere, including the insula, supramarginal gyrus, inferior parietal gyrus, superior temporal gyrus, inferior temporal gyrus, superior temporal sulcus, temporal pole, postcentral gyrus, and fusiform gyrus (Gryglewski *et al*., 2019). Similarly, Sartorius *et al*. (2016) found an increase in CT in the right temporal pole and the bilateral insula gyrus in MDD compared to HC (Sartorius *et al*., 2016). Van Eijndhoven *et al*. (2016) revealed large bilateral clusters of increased CT in TRD after ECT treatment extending from the temporal pole, middle and superior temporal gyrus to the insula, and inferior temporal gyrus in the left hemisphere. Post-hoc analyses demonstrated that the increase in CT of the insular cortex was greater in responders compared to non-responders to ECT. Notably, a higher number of ECT sessions correlated with greater mean CT of the left temporal pole and the left middle and inferior temporal cortex (van Eijndhoven *et al*., 2016). Xu *et al*. (2019) calculated vertex-wise and regional CT within each of the 68 anatomically defined regions from the Desikan–Killiany Atlas in MDD (Xu *et al*., 2019). The authors reported a higher vertex-wise CT in the left temporal pole, left fusiform gyrus, left inferior parietal gyrus, left insula, as well as in the right lateral occipital gyrus, right supramarginal gyrus, right superior temporal gyrus, right insula and right precentral gyrus in patients with MDD after ECT. Furthermore, a greater regional CT was observed in the bilateral fusiform gyrus, bilateral parahippocampus, inferior temporal gyrus, left superior temporal gyrus, left inferior parietal gyrus, left insula, left superior temporal sulcus, right superior frontal gyrus, and the right anterior rostral cingulate, as well as increased vertex-wise and regional CT in the left temporal pole, fusiform gyrus, and bilateral insula in MDD patients after ECT (Xu *et al*., 2019). Pirnia *et al*. (2016) and Yrondi *et al*. (2019) to better delineate the course of CT changes during the entire ECT treatment, acquired an initial scan (T1), an intermediate scan between the first and the last ECT session (T2), and a scan within one week of the last ECT (T3) (Pirnia *et al*., 2016; Yrondi *et al*., 2019). In the study of Yrondi *et al* (2019), the second MRI was performed immediately after the first ECT was considered effective. The researchers detected a significant increase in CT in the superior temporal gyrus in the patient group between baseline and time T3, between T2 and T3, but not between T1 and T2, suggesting that CT changes occur only after multiple ECT sessions. A post-hoc analysis revealed that, at baseline, MDD had thinner CT in the frontal lobe, in the fusiform gyrus, inferior, middle, and superior temporal gyri, parahippocampal gyrus, and the transverse temporal gyrus compared to HC. These differences were no longer significant after ECT (Yrondi *et al*., 2019). In Pirnia *et al*. (2016), the second scan was performed within 48 hours after the second ECT treatment; CT became significantly thicker in the bilateral ACC, right parahippocampal gyrus, superior temporal gyrus, and temporal pole between T1 and T3 in patients with unipolar and bipolar depression. An additional region of interest (ROI) analysis showed significant effects of ECT in the ACC, parahippocampal, entorhinal, superior temporal, inferior temporal, and fusiform cortex. To determine whether ECT was associated with a normalisation towards control values, differences in CT between patients and HC were examined at baseline in the entire cortex and in ROIs that showed significant effects of ECT. Within the cortical ROIs, at baseline, patients showed reduced CT in the fusiform and superior temporal cortex compared to HC, which was not present at the two subsequent time points. Importantly, this study presented a high methodological quality (Pirnia *et al*., 2016). Gbyl *et al*. (2019) explored CT changes in 18 patients with MDD from baseline to six months after the last ECT, with an intermediate scan after the first ECT. They reported an increase in CT in 26 cortical regions, mainly within the frontal, temporal, and insular cortex immediately after the first series of ECT, which returned to baseline at six months of follow-up (Gbyl *et al*., 2019). A recent study by Bracht *et al*. (2023) with high methodological quality compared longitudinal changes in CT between the ECT-treated group, treatment-as-usual (TAU) responders, and HC. Patients with unipolar and bipolar TRD were scanned twice, at the beginning and at the end of the ECT series, while patients with TAU were scanned at baseline and after achieving remission of depressive symptoms or within 12 weeks after the first MRI scan. The global mean CT was increased in the right hemisphere in the ECT group and bilaterally in the TAU responders. Exploratory analyses revealed that CT changes common to the ECT group and TAU-responders included the right insula and the right lateral OFC (Bracht *et al*., 2023). Another recent longitudinal study with high methodological quality explored CT in 96 patients with MDD before and after ECT and reported CT increases in bilateral medial areas of the superior temporal gyrus and bilateral insula compared to baseline (Ji *et al*., 2022). Lastly, Schmitgen *et al*. (2020) investigated several surface-based measures in 12 patients with TRD and found no differences in CT relative to 12 HC and no longitudinal changes of this parameter in the group of patients before and after ECT (Schmitgen *et al*., 2020).

### Correlations between CT changes and clinical improvements

Ten studies explored the correlations between CT changes and clinical improvements in depression measured with HAM-D between baseline and follow-up, and five reported a significant association. Gbyl *et al*. (2019) found a positive correlation between CT increases in the right lateral orbitofrontal gyrus and clinical improvement of depression (Gbyl *et al*., 2019). Similarly, positive correlations were observed between changes in CT in the fusiform, superior, and inferior temporal gyrus and clinical improvement in depression (Pirnia *et al*., 2016); in particular, CT of the ACC increased shortly (T1 and T2) after the initiation of ECT treatment and was predictive of the general clinical response (>50% improvement in HAM-D scores) to ECT. In van Eijndhoven *et al*. (2016), increased mean CT of the right insula was associated with clinical improvement in depressive symptoms (van Eijndhoven *et al*., 2016). Schmitgen *et al*. (2020) observed that pre- and post-ECT CT of the left rostral anterior cingulate gyrus predicted a greater improvement in depression. Furthermore, the authors also found a correlation between clinical improvement and increased CT of the left medial orbitofrontal gyrus after but not before ECT treatment, suggesting a mediator role of cortical changes in this region (Schmitgen *et al*., 2020). Lastly, Xu *et al*. (2019) reported a correlation between vertex-wise CT increases in the left occipital gyrus and the improvement of depression. Furthermore, to test the brain mechanisms of tolerability of ECT, the authors explored the association between cognitive functions and CT changes. In particular, ECT-dependent memory impairments measured by Auditory Verbal Learning Test (ALVT) delayed recall scores were negatively correlated with CT increase in the left inferior parietal gyrus and marginally in the right precentral gyrus after ECT treatment (Xu *et al*., 2019).

## Discussion

This review summarised the evidence on CT changes associated with ECT among patients with depression. We found that ECT was associated with a widespread increase in CT in patients with depression, located primarily in the temporal lobe and insula cortex and, to a lesser extent, in the frontal areas. This pattern of increased CT was consistent across studies with lower and higher methodological quality scores. Some investigations showed a correlation between increases in CT and clinical improvement, but this was only confirmed by a few studies with higher methodological quality.

### Temporal lobe

Most of the reviewed studies found that ECT was followed by increased CT in the temporal lobe, including the superior, middle, and inferior temporal gyri and temporal pole (Pirnia *et al*., 2016; Sartorius *et al*., 2016; van Eijndhoven *et al*., 2016; Gbyl *et al*., 2019; Gryglewski *et al*., 2019; Xu *et al*., 2019; Yrondi *et al*., 2019; Ji *et al*., 2022). Although most increases were observed near the placement of the electrodes, temporal changes were also observed when the electrodes were located in the frontal lobe (Xu *et al*., 2019; Ji *et al*., 2022). Despite decades of use, the precise mechanisms by which ECT exerts its therapeutic effects remain poorly understood (Fridgeirsson *et al*., 2021; Deng *et al*., 2023). Among the hypotheses of ECT action, three main theories have been proposed: the generalised seizure theory, the neuroendocrine–diencephalic theory, and a combined anatomical–ictal theory (Bolwig, 2011). According to the generalised seizure theory, the therapeutic effects of ECT depend on the elicitation of generalised seizures. Indeed, as first demonstrated by Ottosson in 1960, generalised seizures are essential for the efficacy of ECT, and subconvulsive stimuli have weaker or no antidepressant effect (Ottosson, 1960). This theory is supported by evidence showing that bilateral electrode placement, which induces a more pronounced seizure generalisation, presents a higher therapeutic efficacy compared to unilateral electrode placement (Bolwig, 2011). In addition, seizure-inducing drugs, such as flurothyl and pentylendetetrazol, show similar clinical effects to ECT (Karliner and Padula, 1960; Cooper and Fink, 2014). However, recent research has challenged the notion that widespread seizure activity is the main responsible for ECT efficacy in depression, given the evidence that increasing the strength of the electric field can affect the clinical effectiveness of ECT, without affecting the characteristics of the seizure (Argyelan *et al*., 2019). Notably, current literature suggests a higher intensity of the electric field may be associated with greater neuronal plasticity after treatment (Argyelan *et al*., 2019). In this regard, animal studies have shown that electroconvulsive seizures stimulate dendritic arborisation, synaptic density, and glial proliferation (Ongür *et al*., 2007; Maynard *et al*., 2018). Furthermore, a recent quantitative MRI study that explored brain’s myelin, iron, and tissue water content at multiple time-points before, during and after ECT treatment, suggested that myelin changes rather than oedema are associated with the longitudinal effects of ECT (Gyger *et al*., 2021). This may be a consequence of the increased expression and release of BDNF induced by ECT (Polyakova *et al*., 2015), which could ultimately be the basis for the therapeutic efficacy of ECT. Taken together, this evidence seems to support the hypothesis that seizure activity synergistically with an electric field is involved in the clinical efficacy of ECT and has a profound effect on the biology of the human brain. Unfortunately, only two of the included studies reported information about seizure duration (Gryglewski *et al*., 2019; Van Eijndhoven *et al*., 2016) and no studies described the electric field strength, so we cannot draw any conclusion on the association between ECT-induced seizure activity or ECT-induced electric field, CT changes, and antidepressant effect.

The temporal lobe plays a crucial role in the pathophysiology of depression, as it is involved in the core neuropsychological features that are commonly disrupted in MDD and bipolar depression, including emotional processing and social cognition (Takahashi *et al*., 2010; Gillissie *et al*., 2022). Interestingly, cortical abnormalities in the temporal lobe have been commonly described in MDD and BD (Schmaal *et al*., 2017; Hibar *et al*., 2018). Furthermore, an accumulating body of evidence shows that the neurobiology of depression involves structural and functional dysfunctions in the corticolimbic network, particularly in the temporal lobe, hippocampus, and amygdala (Zhong *et al*., 2016; Tassone *et al*., 2022). Recently, Leaver *et al*. (2022) hypothesised a “network mechanism” of ECT, according to which seizures originating from the middle temporal lobe improve depressive symptoms by correcting or resetting corticolimbic alterations (Leaver *et al*., 2022). It has been suggested that the increase in temporal CT after ECT could be due to brain damage, including reactive inflammation or oedema due to the electrical current near the site of the electrode application (Coffey *et al*., 1991); thus, an increase in temporal CT after ECT could be considered a transient epiphenomenon of treatment, rather than a mechanism underlying its efficacy (Puri *et al*., 1998). However, although some accumulation of extracellular fluid has been observed using diffusion tensor imaging (Repple *et al*., 2020), most studies have not detected post-ECT gliosis or brain oedema (Szabo *et al*., 2007), and specific studies, using T2-relaxometry to detect significant fluid shifts, did not support oedema as a cause of structural changes detected after ECT (Girish *et al*., 2001; Kunigiri *et al*., 2007; Yrondi *et al*., 2018). Furthermore, a systematic review and meta-analysis of the effects of ECT on brain volumes (Gbyl and Videbech, 2018) did not support the theory of brain damage and did not find an increase in brain damage markers (Zachrisson *et al*., 2000; Agelink *et al*., 2001; Kranaster *et al*., 2014). These findings were confirmed by Gylb et al. (2022), who found no differences in serum S100B, a marker of brain injury, after an ECT series (Gbyl *et al*., 2022). Crucially, cortical thickening in the temporal lobe in MDD has been previously reported in association with antidepressant drug treatment (Nemati and Abdallah, 2020), suggesting that these effects could be a common mechanism underlying the effectiveness of antidepressant treatments. Consistent with the theories of neurotrophic and neuroplasticity of depression (Duman and Li, 2012), the observed changes in CT during ECT treatment may be the result of underlying increased neural growth, synaptogenesis, and synaptic rearrangement. This is in line with an increase in neurotrophins after ECT (Yrondi *et al*., 2018).

Increased CT in the superior and inferior temporal gyrus was associated with an improvement in depressive symptoms only in one study (Pirnia *et al*., 2016). Furthermore, Gbyl *et al*. (2019) observed that, although CT in the temporal lobe increased after the first ECT, it returned to baseline after six months, even if the antidepressant effect persisted (Bolwig, 2011).

Taken together, this evidence suggests that temporal CT is primarily affected by ECT, regardless of electrode placement. Less clear is the role of temporal CT changes in clinical improvements. Although most investigations showed that changes in CT did not correlate with clinical symptoms, this was not replicated in all included studies. Therefore, more evidence is needed to clarify the role of temporal CT in changes in depressive symptoms.

### Insula

Seven investigations reported an increase in CT in the insula after ECT (Pirnia *et al*., 2016; Sartorius *et al*., 2016; van Eijndhoven *et al*., 2016; Gbyl *et al*., 2019; Gryglewski *et al*., 2019; Xu *et al*., 2019; Ji *et al*., 2022). Four of these studies presented the highest methodological scores. Previous literature has shown that the insula contributes to emotion regulation and pain perception and is also involved in the pathophysiology of MDD and bipolar depression (Nagai *et al*., 2007; Sprengelmeyer *et al*., 2011; Schmaal *et al*., 2017; Qiu *et al*., 2020). A study showed a correlation between cortical thickening in this region and reduced severity of depression (van Eijndhoven *et al*., 2016). Interestingly, Bracht *et al*. (2023) found an increase in CT in the right insula in both ECT and TAU responders, suggesting a potential role for CT as a marker of treatment response, which may not be specific for ECT (Bracht *et al*., 2023). In addition, recent evidence has shown that insular metabolic activity may represent a potential predictive biomarker of remission in MDD after treatment with antidepressants or psychotherapy (Kelley *et al*., 2021). Overall, this literature suggests that structural and functional insular abnormalities may play a role as biomarkers of antidepressant effects and predictive biomarkers of symptom remission, regardless of the type of treatment. In summary, the study results suggest that, although ECT is associated with an increase in insular CT, the improvement in depression symptoms after ECT does not appear to be associated with CT changes in the insula.

### Frontal lobe

The frontal lobe, in particular the OFC, the superior, middle, and inferior frontal gyrus, have been consistently implicated in the pathophysiology of unipolar and bipolar depression (Drevets, 2007; Altshuler *et al*., 2008; Fitzgerald *et al*., 2008). Three of the ten studies included in the present review showed cortical thickening in these areas (Gbyl *et al*., 2019; Xu *et al*., 2019; Bracht *et al*., 2023) and, interestingly, only in the study by Xu *et al*. (2019) the electrodes were localised in the frontal lobe. Remarkably, Gbyl *et al*. (2019) reported a strong association between cortical thickening of the right lateral OFC and the antidepressant effect of ECT (Gbyl *et al*., 2019). In depression, a reduced volume along with altered activity in OFC has previously been reported (Drevets, 2007). A recent study showed that direct stimulation of this region produced an acute improvement in depressive symptoms in epileptic subjects with moderate to severe depression (Rao *et al*., 2018). Noticeably, OFC CT increased immediately after the ECT series but returned to baseline levels at six months of follow-up, despite the continued antidepressant effect, suggesting that the persistence of the clinical efficacy of ECT was not related to orbitofrontal CT. Interestingly, a recent GEMRIC consortium study identified a multivariate discriminative pattern of volume alterations in the cortical midline, striatal, and lateral prefrontal areas that differentiated responders from non-responders, suggesting that structural changes in prefrontal areas might play a role in the clinical response to ECT (Mulders *et al*., 2020).

### Other brain areas

ECT was associated with cortical thickening in several other areas, including the ACC, the parahippocampal gyrus, the entorhinal cortex, the fusiform gyrus, the superior and inferior parietal gyrus, the pre- and postcentral gyrus, and the lateral occipital gyrus. Although all these areas have been consistently implicated in the pathogenesis of unipolar and bipolar depression and appear to be influenced by antidepressant treatments (Bartlett *et al*., 2018; Klimes-Dougan *et al*., 2018; Yi *et al*., 2022), only increased CT in the right lateral occipital gyrus, an area involved in the perception of the face and emotion (Nagy *et al*., 2012), was associated with clinical improvement (Xu *et al*., 2019). Future studies with larger sample sizes are needed to clarify the effect of ECT on CT in these areas and the potential association with clinical improvements.

### Biological significance of CT findings

Cortical thickening has been previously reported in patients with depression after antidepressant medications (Bartlett *et al*., 2018; Koenig *et al*., 2018; Krause-Sorio *et al*., 2020) and neurostimulation treatments (Phillips *et al*., 2015; Dalhuisen *et al*., 2021; Wu *et al*., 2022), suggesting that cortical changes may not be specifically associated with ECT, but rather represent a generally shared mechanism of effectiveness of various antidepressant treatments. Surprisingly, however, both pretreatment CT and the early increase in CT seem to represent predictive biomarkers of the clinical response to antidepressants (Phillips *et al*., 2015), suggesting that various processes can underlie its clinical effectiveness. Neuroplasticity, including the processes of neurogenesis, synaptogenesis, dendrogenesis, gliogenesis, and angiogenesis, has been hypothesised to be the biological mechanism for the effects of ECT on CT (Wennström *et al*., 2004; Hellsten *et al*., 2005; Jaggar *et al*., 2023; Zhang *et al*., 2023). In particular, animal studies have reported that rats subjected to electroconvulsive stimulation show increased genesis of hippocampal neurons with the potential for long-term survival (Altar *et al*., 2003; Madsen *et al*., 2005; Olesen *et al*., 2017). In humans, ECT is associated with a rapid and widespread increase in GM volume in the post-treatment phase (Ousdal *et al*., 2020), as well as an increased BDNF and vascular endothelial growth factor (VEGF) (Vanicek *et al*., 2019; Sorri *et al*., 2021). However, the promptness of these volumetric changes suggests that neurogenesis is unlikely to be the only mediating factor for the volumetric effects of ECT (Ousdal *et al*., 2022). Taking into account this evidence, Ousdal *et al*. (2022) conceptualised a time-dependent model of the neural effects of ECT that includes three consecutive phases: disruption as the immediate effect, increased neuroplasticity as the short-term effect, and rewiring as the long-term effect (Ousdal *et al*., 2022). Most of the studies included in our review examined the short-term mechanisms of ECT. The only investigation that evaluated the long-term effects of ECT reported that CT returned to baseline levels after 6 months (Gbyl *et al*., 2019). Although these results seem to indicate that CT is more sensitive in detecting immediate changes associated with brain atrophy (Lemaitre *et al*., 2012) compared to GM volume morphometry that can better measure neuroplasticity and rewiring processes after electrical stimulation (Argyelan *et al*., 2019), this study only included 14 patients, so the results should be interpreted with caution. Importantly, the findings of broad cortical thickening after ECT are in line with volumetric studies (Ousdal *et al*., 2022). Interestingly, two of the included studies explored GM volume in addition to CT (Sartorius *et al*., 2016; Gryglewski *et al*., 2019). Although Gryglewski *et al*. (2019) focused only on hippocampal and amygdala volume (Gryglewski *et al*., 2019), Sartorius *et al*. (2016) examined whole-brain GM volume and CT changes and showed a pattern of similar abnormalities involving the temporal lobe and the insula (Sartorius *et al*., 2016), suggesting that CT and GM volume may provide complementary information that could help to understand the mechanisms underlying the differential changes in the brain across the cortical regions after ECT.

### Limitations and conclusions

The findings of this review should be interpreted in light of the limitations of primary studies. First, the small sample size of the included studies could have skewed the results and decreased the power of the studies, leading to a type II error. Thus, the abnormalities described in this review might be limited to some brain areas that presented changes with large effect sizes, neglecting changes with smaller effect sizes. This could, at least partially, explain why the mega-analysis by Ousdal *et al*. (2020) showed more widespread ECT-related brain changes (Ousdal *et al*., 2020). Furthermore, only six studies had a control group, and not all studies reported baseline differences in CT between patients and HC. Second, the different clinical phenotypes of the participants (e.g., comorbidities, illness phase, duration of the illness) may have contributed to the heterogeneity of the results. In addition, two studies included individuals with bipolar depression, which may have affected their results. Third, the studies did not report effect size but only significance levels. The significance of a result depends on both effect and sample size, so that with a small effect and few individuals, a low number of regions can be detected. Fourth, the heterogeneity in the positioning of the electrodes during the administration of the ECT may have also influenced CT results. Similarly, the timing of the second MRI scan was not clearly defined in all studies, leading to the speculation that some studies conducted the second MRI immediately after ECT and others completed it some days after ECT, which could have affected the CT findings. Lastly, in this literature, the sex-related difference in CT changes after ECT was not investigated, and this could be due to the lack of evidence of sex differences in CT in depression.

In conclusion, available studies lend support to the hypothesis that ECT in depression is associated with cortical thickening of the temporal lobe, insula, and, to a lesser extent, frontal lobe, and several other areas of the brain involved in the pathophysiology of depression. These findings are consistent with evidence of increased CT after pharmacological and neuromodulation treatments, suggesting that cortical changes may not be specific to ECT, but rather reflect mechanisms of therapeutic response. Future studies with higher methodological quality, longer follow-up periods, and multiple methods of evaluating morphological changes are warranted to increase knowledge of the effects of ECT on CT in MDD. Such studies may also clarify the potential role of CT and other cortical measures, including surface area, gyrification, and cortical complexity, as biomarkers of the clinical response in depression.

## Supporting information

Toffanin et al. supplementary material Toffanin et al. supplementary material

## Supplementary material

The supplementary material for this article can be found at https://doi.org/10.1017/neu.2024.6.
