# Supplementary material for: Effects of electroconvulsive therapy on cortical thickness in depression: a systematic review
Source: Acta Neuropsychiatr. 2024 Feb 12;37:e44. doi: 10.1017/neu.2024.6 (PMC13130300; doi:10.1017/neu.2024.6)
Supplement: Toffanin et al. supplementary material [file S0924270824000061sup001.docx]

**Effects of electroconvulsive therapy on cortical thickness in depression: A systematic review**

*Supplementary Material*

*Assessment of methodological quality*

The methodological quality of the included studies was assessed using a point system derived from Gbly and Videbech (2018) (Gbyl and Videbech, 2018). The quality score consists of the following items: a) number of subjects (0,1 point was assigned for every subject enrolled); b) the presence of a control group (1 point was assigned for the presence of an age- and gender-matched group of healthy controls, additional 1 point for the presence of a control group of depressed patients not treated with ECT); c) the number of MRI scans the control group underwent (1 point assigned if the group was scanned twice or more); d) MRI scanner field strength (1 point for 3 Tesla); e) voxel size (1 point given for voxel size lower than 1.0 mm^3^; f) medication status (1 point if subjects were not medicated or if medication had been washed out in >80% of subjects before inclusion); g) consecutively collected sample (1 point was assigned if it was explicitly stated that a sample was collected in a consecutive way); h) duration of follow-up time (1 point for every MRI scan conducted later than 2 weeks after completion of the ECT series). The higher the score, the better the methodological quality.

**Table S.1. Assessment of the methodological quality of the included studies.**

| **Study** | **N° of subjects** | **Control group** | **N° of MRI scans in the control group** | **MRI scanner field strength** | **Voxel size** | **Medication status** | **Consecutively collected sample** | **Duration of follow-up** | **Total quality score** |
| --- | --- | --- | --- | --- | --- | --- | --- | --- | --- |
| Pirnia et al., 2016 | 2.9 | 1 | 1 | 1 | 0 | 1 | 0 | 0 | 6.9 |
| Sartorius et al., 2016 | 1.8 | 1 | 0 | 1 | 0 | 0 | 0 | 0 | 3.8 |
| Van Eijndhoven et al., 2016 | 2.3 | 1 | 1 | 0 | 0 | 1 | 0 | 0 | 5.3 |
| Gbyl et al., 2019 | 1.8 | 0 | 0 | 1 | 1 | 0 | 0 | 1 | 4.8 |
| Gryglewski et al., 2019 | 1.4 | 0 | 0 | 1 | 0 | 0 | 0 | 0 | 2.4 |
| Schmitgen et al., 2019 | 1.2 | 1 | 0 | 1 | 0 | 0 | 0 | 0 | 3.2 |
| Xu et al., 2019 | 2.3 | 0 | 0 | 1 | 0 | 0 | 0 | 0 | 3.3 |
| Yrondi et al., 2019 | 1.7 | 1 | 1 | 1 | 0 | 0 | 0 | 0 | 4.7 |
| Ji et al., 2022 | 5.6 | 1 | 1 | 1 | 0 | 0 | 0 | 0 | 8.6 |
| Bracht et al., 2023 | 2.0 | 2 | 1 | 1 | 0 | 0 | 0 | 0 | 6 |

**Table S.2. Clinical scales scores in the included subjects.**

| **Author, year** | **Subjects (M/F)** | **Clinical scales scores** |
| --- | --- | --- |
| Pirnia et al., 2016 | Patients: 29 (11/18) | **T1-T2-T3**  HAM-D: 26.3 (4.93); 20.5 (6.47); 12.2 (8.26)  MADRS: 40.6 (7.57); 32.6 (8.67); 15.6 (11.4) |
|  | HC: 29 (13/16) | NA |
| Sartorius et al., 2016 | Patients: 18 (9/9) | HAM-D _21_  Pre-ECT: 31.8 (8.2)  Post-ECT: 10.67 (7.3) |
|  | HC:36 (18/18) | NA |
| Van Eijndhoven et al., 2016 | Patients: 23 (8/15) | HAM-D_17_  Pre-ECT: 21.9 (5.3)  Post-ECT: 12.6 (7.1) |
|  | HC: 22 (8/14) | NA |
| Gbyl et al., 2019 | Patients: 18 (8/10) | **T1-T2-T3**  HAM-D_17_: 29.4 (95% CI: 26.6,32.3); 9.7 (95% CI: 6.8,12.5); 10.7 (95% CI: 7.6, 13.8)  HAM-D_6_: 14.2 (95% CI: 12.6,15.8); 5.1 (95% CI: 3.4, 6.7); 5.2 (95% CI: 3.4, 7.0) |
| Gryglewski et al., 2019 | Patients: 14  (3/11) | HAM-D _17_  Pre-ECT: 25.4 (3.3)  Post-ECT: 7.1 (4.2) |
| Schmitgen et al., 2019 | Patients: 12 (4/8) | HAM-D _17_  Pre-ECT: 26.8 (6.5)  Post-ECT: 6.5 (3.9) |
|  | HC: 12 (4/8) | NA |
| Xu et al., 2019 | Patients: 23 (11/12) | HAM-D _17_  Pre-ECT: 22.2 (4.7)  Post-ECT: 3.8(2.2) |
| Yrondi et al., 2019 | Patients: 17 (NR) | NR |
|  | HC: 24 (NR) | NA |
| Ji et al., 2023 | Patients: 56 (12/44) | HAM-D  Pre-ECT: 24.0 (6.3)  Post-ECT: 6.8 (5.6) |
|  | Patients: 40 (13/27) | HAM-D  Pre-ECT: 22.5 (4.3)  Post-ECT: 5.4 (4.8) |
| Bracht et al., 2023 | Patients ECT: 20 (12/8) | HAM-D _21_  Pre-ECT: 21.4 (5.3)  Post-ECT: 10.9 (8.1)  BDI-II  Pre-ECT: 30.6 (8.1)  Post-ECT: 20.8 (10.3) |
|  | Patients TAU: 20  (6/14) | HAM-D _21_  Baseline: 22.6 (5.1)  Follow-up: 5.6 (3.2)  BDI-II  Baseline: 26.8 (8.8)  Follow-up: 13.1(8.4) |
|  | HC: 20 (12/8) | HAM-D _21_  Baseline: 0.65 (1.0)  Follow-up 0.25 (0.8)  BDI-II  Baseline: 1.5 (2.4)  Follow-up: 1 (1.8) |

BDI-II: Beck depression inventory-II; ECT: electroconvulsive therapy; HAM-D: Hamilton rating scale for depression; HC: healthy controls; MADRAS: Montgomery-Åsberg depression rating scales; NR: not reported; NA: not applicable; NR: not reported; TAU: treatment as usual; TRD: treatment resistant depression.

**Table S.3. Statistical analyses in the included studies.**

| **Author, year** | **Statistical analyses** |
| --- | --- |
| Pirnia et al., 2016 | Significance was set at p < 0.005 with FDR correction for multiple comparisons. |
| Sartorius et al., 2016 | A threshold of p = 0.05 FWE corrected on cluster level with a cluster building threshold of p = 0.001 uncorrected was used. |
| Van Eijndhoven et al., 2016 | Correction for multiple comparisons was applied by clusterwise correction, based on Monte Carlo Z simulation (threshold 0.005) |
| Gbyl et al., 2019 | Significance was set at p < 0.05 with the Benjamini-Hochberg FDR correction for multiple comparisons. |
| Gryglewski et al., 2019 | Bonferroni correction for FWE by multiplying p-values with the number of regions (34 for cortical areas) was used. Results were considered significant at an FWE-corrected probability of type I error of α ≤ 0.05. Trend-level effects were reported after controlling with the Benjamini-Hochberg FDR correction for multiple comparisons at a level of p < 0.05. |
| Schmitgen et al., 2019 | Significance was set at p < 0.05 uncorrected. |
| Xu et al., 2019 | - Vertex-wise SBM analyses: results were corrected for multiple comparisons using Monte Carlo simulation (a pre- cached cluster-wise level of p < 0.05, a voxel-wise level of p < 0.001). - Regional CT analyses: a Bonferroni correction with p < 0.05/68 was used. |
| Yrondi et al., 2019 | Significance was set at p < 0.005 with FDR correction for multiple comparisons. |
| Ji et al., 2023 | Significance was set at p < 0.005 with false FDR correction for multiple comparisons. |
| Bracht et al., 2023 | All tests were two-tailed and a level of significance of p < 0.05 was applied. |

BDI-II: Beck depression inventory-II; CT: cortical thickness; ECT: electroconvulsive therapy; FDR: false discovery rate; FEW: family-wise error; HAM-D: Hamilton rating scale for depression; HC: healthy controls; MADRAS: Montgomery-Åsberg depression rating scales; NR: not reported; NA: not applicable; NR: not reported; SBM: surface-based morphometry; TAU: treatment as usual; TRD: treatment resistant depression.
